# Predictive Factors for Successful Decannulation in Patients with Tracheostomies and Brain Injuries: A Systematic Review

**DOI:** 10.1007/s00455-023-10646-2

**Published:** 2024-01-08

**Authors:** Thomas Gallice, Emmanuelle Cugy, Olivier Branchard, Patrick Dehail, Geoffroy Moucheboeuf

**Affiliations:** 1 Neurosurgery Unit B, Bordeaux University Hospital, Pellegrin Hospital, 33000 Bordeaux, France; 2 Neurological ICU, Bordeaux University Hospital, Pellegrin Hospital, 33000 Bordeaux, France; 3 Physical and Rehabilitation Medicine Unit, Swallowing Evaluation Unit, Bordeaux University Hospital, Tastet-Girard Hospital, 33000 Bordeaux, France; 4 Bordeaux Research Center for Population Health (BPH), Team: ACTIVE, University Bordeaux Segalen, UMR_S 1219, 33000 Bordeaux, France; 5 Physical and Rehabilitation Medicine Unit, Bordeaux University Hospital, Tastet-Girard Hospital, 33000 Bordeaux, France; 6 Traumatic and Surgical ICU, , Bordeaux University Hospital, Pellegrin Hospital, 33000 Bordeaux, France; 7 Physical and Rehabilitation Medicine Unit, Arcachon Hospital, 33260 La Teste de Buch, France

**Keywords:** Acquired brain injury, Decannulation, Predictive factors, Rehabilitation, Tracheostomy weaning

## Abstract

Neurological patients frequently have disorders of consciousness, swallowing disorders, or neurological states that are incompatible with extubation. Therefore, they frequently require tracheostomies during their stay in an intensive care unit. After the acute phase, tracheostomy weaning and decannulation are generally expected to promote rehabilitation. However, few reliable predictive factors (PFs) for decannulation have been identified in this patient population. We sought to identify PFs that may be used during tracheostomy weaning and decannulation in patients with brain injuries. We conducted a systematic review of the literature regarding potential PFs for decannulation; searches were performed on 16 March 2021 and 1 June 2022. The following databases were searched: MEDLINE, EMBASE, CINAHL, Scopus, Web of Science, PEDro, OPENGREY, OPENSIGLE, Science Direct, CLINICAL TRIALS and CENTRAL. We searched for all article types, except systematic reviews, meta-analyses, abstracts, and position articles. Retrieved articles were published in English or French, with no date restriction. In total, 1433 articles were identified; 26 of these were eligible for inclusion in the review. PFs for successful decannulation in patients with acquired brain injuries (ABIs) included high neurological status, traumatic brain injuries rather than stroke or anoxic brain lesions, younger age, effective swallowing, an effective cough, and the absence of pulmonary infections. Secondary PFs included early tracheostomy, supratentorial lesions, the absence of critical illness polyneuropathy/myopathy, and the absence of tracheal lesions. To our knowledge, this is the first systematic review to identify PFs for decannulation in patients with ABIs. These PFs may be used by clinicians during tracheostomy weaning.

**Supplementary Information:**

The online version contains supplementary material available at 10.1007/s00455-023-10646-2.

## Introduction

During their stay in an intensive care unit (ICU), approximatively 10% of patients will require tracheostomies [1]. The clinical scenarios that are most likely to require tracheostomies include reduction of dead space during mechanical ventilation weaning, treatment of a consciousness disorder, treatment involving a neurological status that is incompatible with extubation, the need to counteract inefficient airway protection related to a neurological lesion and/or to ICU hospitalisation [1–3]. Tracheostomy weaning and its final step, decannulation, is a complex process. The procedure requires a weaning protocol and a trained multidisciplinary team (an intensivist, an ear nose and throat (ENT) specialist, physiotherapists, speech therapists, and nurses); it can be performed in the ICU or after ICU discharge [4–6]. Weaning protocols usually involve decision-making procedures that are based on clinical criteria and sometimes instrumental assessments [7–10]. However, many of these protocols heavily rely on expert opinion, and there are limited evidence-based guidelines for successful decannulation [2].

A systematic review by Santus et al. concluded that a strong cough and the ability to tolerate tube capping are predictive factors (PFs) for decannulation [3]. The same review described secondary PFs that may also be important. These secondary PFs included the level of oxygenation, capnia, the level of consciousness and neurological state, age, swallowing status, the quantity and quality of secretions, the duration of mechanical ventilation, the stability of haematic gases (PaO_2_ and PaCO_2_), the aetiology of respiratory failure, and comorbidities [3]. A literature review by Meideros et al. concluded that the following criteria were indicators for decannulation success: clinical and haemodynamic stability, high level of alert consciousness, no requirement for mechanical ventilation, no dependence on humidification, good secretion management, and the absence of bronchoaspiration [4]. Meideros et al. reported that swallowing, airway patency, and secretion management assessments were important steps in tracheostomy weaning [4].

However, these previous reviews included patients who had undergone tracheostomies for various reasons (e.g., respiratory, neurological, and swallowing disorders). In contrast, patients with acquired brain injuries (ABIs) may require tracheostomies for the treatment of a particular neurological defect [5]. Therefore, the factors that influence tracheostomy weaning and decannulation in such cases may be unique to this patient population. Here, we sought to identify PFs that may be used by clinicians to predict success, failure, or difficulties during tracheostomy weaning and decannulation. Validated PFs for these procedures will enable clinicians to provide better treatment.

## Materials and Methods

We used the Population–Intervention–Control–Outcome (PICO) research strategy.

- Population: All patients were aged > 18 years and had ABIs from traumatic, vascular, encephalopathic, or oncological aetiologies. Patients were required to be free from mechanical ventilation, and articles describing patients with medullar lesions or neurodegenerative diseases were excluded.
- Intervention: Decannulation and/or tracheostomy weaning.
- Control: none.
- Outcome: PFs for decannulation success and/or failure and/or delayed and/or difficult and/or easy tracheostomy weaning.

To identify PFs, we evaluated sociodemographic (age and sex) and clinical data, aetiologies, ABI treatments, disease severity, related disabilities (cognitive and functional), comorbidities, medical and surgical history, and variables linked to tracheostomies (e.g., SpO_2_ level, tube capping tolerance, and tracheostomy timing).

Interventions such as specific rehabilitation strategies (e.g., electric stimulation), specific assessments (e.g., use of flexible bronchoscopy), and specific weaning procedures (e.g., use of a team-based procedures) were not regarded as potential PFs. We searched for all types of articles, except for systematic reviews, meta-analyses, abstracts, and position articles. Eligible articles were published in English or French. Our principal outcome was the identification of PFs for successful or failed decannulation. Our secondary outcome was the identification of PFs for delayed decannulation or PFs for difficult or easy tracheostomy weaning.

We searched the following electronic databases: MEDLINE, EMBASE, CINAHL, Scopus, Web of Science, PEDro, OPENGREY, OPENSIGLE, Science Direct, CLINICAL TRIALS and CENTRAL. There were no restrictions on the date of publication. The first search was performed on 16 March 2021 (deadline, 16 March 2021). Searches were repeated on 1 June 2022 (with a date filter of 16 March 2021 or the 2021–2022 period, depending on the database), immediately prior to the final analyses and hand searching of the bibliographies.

- On MEDLINE, we searched (tracheostom* [MH] or tracheostom* [TIAB] or tracheostom*[OT] or tracheotom*[MH] or tracheotom*[TIAB] or tracheotom*[OT] or cannula*[MH] or cannula*[TIAB] or cannula*[OT]) and (brain injur*[MH] or brain injur*[TIAB] or brain injur*[OT] or stroke [MH] or stroke [TIAB] or stroke [OT] or traumatic brain injur* [MH] or traumatic brain injur* [TIAB] or traumatic brain injur*[OT] or neuro* [MT] or neuro* [TIAB] or neuro* [OT] or neuro* or central nervous system [MT] or central nervous system [TIAB] or central nervous system [OT]) and (weaning or weaning [TIAB] or weaning [OT] or decannulation or decannulation [TIAB] or decannulation [OT] or tube removal or tube removal [TIAB] or tube removal [OT])
- On EMBASE, we searched «tracheostomy and decannulation and stroke or brain injury»
- On CINAHL, we searched «tracheostomy and decannulation and brain injury or post-stroke or stroke or head injury or traumatic brain injury or post-stroke or stroke or head injury or traumatic brain injury or acquired brain injury»
- On CENTRAL, we searched «tracheostomy and decannulation and stroke or brain injury»
- On OPENGREY/OPENSIGLE, we searched «tracheostomy and decannulation»
- On PEDro, we searched «tracheostomy and decannulation»
- On Web of Science, we searched «tracheostomy and decannulation»
- On CLINICAL TRIALS, we searched «tracheostomy and decannulation»
- On Scopus, we searched «tracheostomy and decannulation and (brain injury or stroke or CNS)»
- On Science Direct, we searched «tracheostomy and decannulation and (brain injury or stroke or CNS)»

Articles were entered into the Rayyan QCRI data management and extraction web application (http://rayyan.qcri.org). Duplicates were removed, and two reviewers (TG and GM) screened articles independently. Initial selection was based on the assessment of each article’s title and abstract. If necessary, the entire article was read. Disagreements regarding article inclusion were resolved by discussion. Remaining disagreements were resolved by consensus, in consultation with a third reviewer (EC). The reasons for exclusion of each article were recorded.

Risk of bias (RoB) assessment was performed for each article by TG and GM independently, using the Quality in Prognosis Study (QUIPS) tool [6]. Consensus was sought between the two reviewers, and any disagreements were resolved by consultation with a third reviewer (EC). The QUIPS tool has a reported interrater agreement ranging between 70% and 89.5% (median 83.5%) [7]. This search strategy was registered on PROSPERO on 8 April 2021 (CRD42021246999).

## Results

**Fig. 1:**
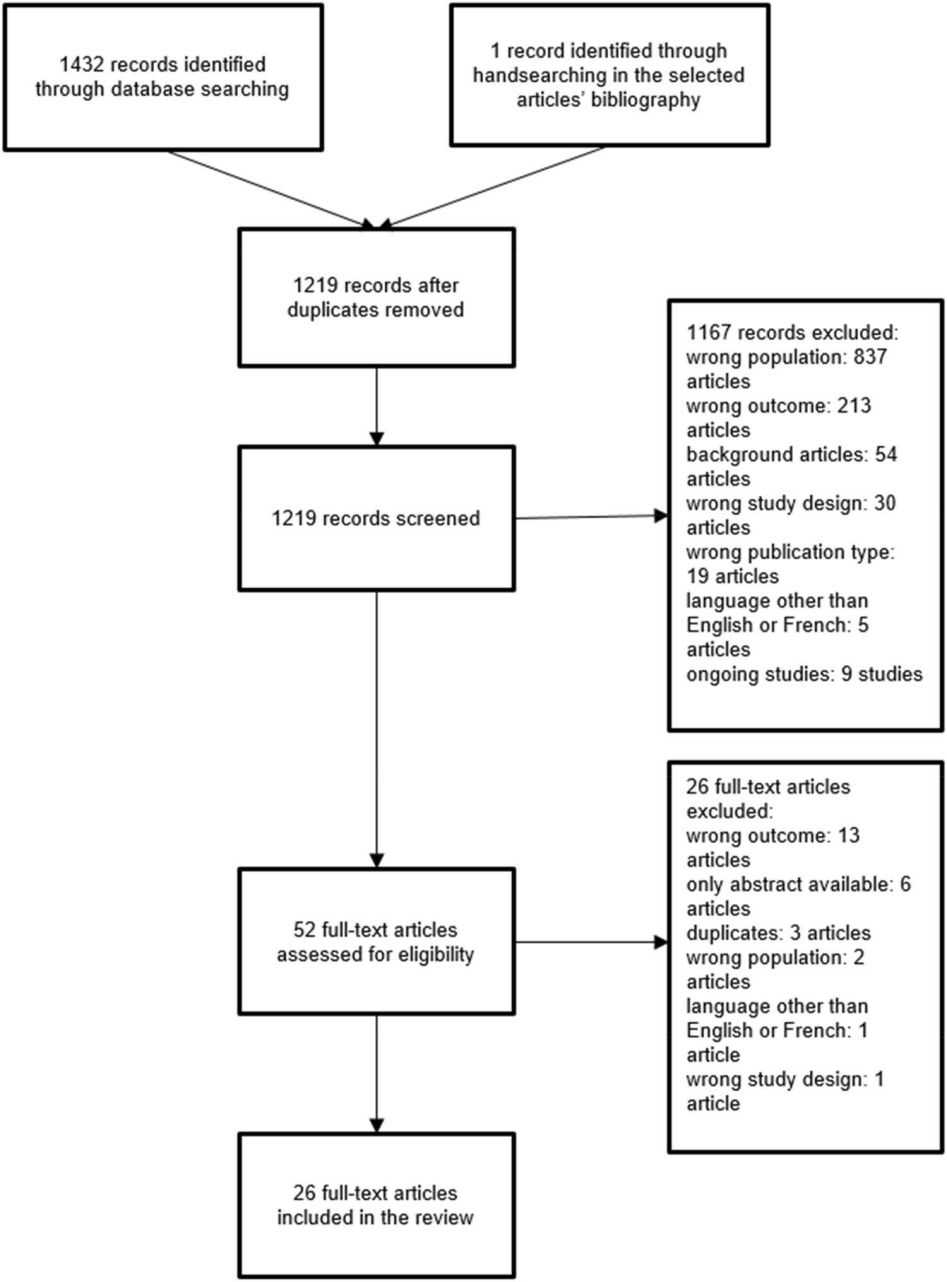
Flow chart

In total, 1433 articles were identified, and 214 duplicates were removed. Of the remaining 1219 articles, 1167 were excluded by screening. The reasons for exclusion were wrong population (837 articles), wrong outcome (213 articles), background articles (54 articles), wrong study design (30 articles), wrong publication type (19 articles), publication in a language other than English or French (5 articles: 1 in German, 1 in Japanese, and 3 in Mandarin Chinese), and ongoing investigation (9 articles). The full-text versions of 52 articles were assessed for eligibility, and 26 articles were excluded. The reasons for exclusion were wrong outcome (13 articles), publication as an abstract only (6 articles), duplicate publication (3 articles), wrong population (2 articles), publication in a language other than English or French (1 article in Mandarin Chinese), and wrong study design (1 article). Therefore, 26 full-text articles were included in this review (Fig. 1); 5 described patients with traumatic brain injuries (TBIs), 17 described patients with ABIs (various aetiologies), and 4 described patients with stroke.

There were two types of study design. In total, 9 studies assessed a predefined PF (7 for patients with ABIs, 1 for patients with TBIs, and 1 for patients with stroke), whereas 17 studies sought to identify PFs among many variables (10 for patients with ABIs, 4 for patients with TBIs, and 3 for patients with stroke). One of these 26 articles described a population of patients with ABIs but included a subgroup analysis for patients with stroke (Reverberi et al., 2018). Consequently, this study is described in both the ABI and stroke subgroups [20].

Among the 26 included studies, there were 15 retrospective monocentric observational studies, 8 prospective monocentric observational studies, 1 retrospective monocentric case–control observational study, 1 retrospective multicentric observational study, and 1 prospective multicentric observational study. According to the QUIPS tool, 10 articles had a low RoB, 10 articles had a moderate RoB, and 6 articles had a high RoB. Our interrater agreement was 65% after the first blinded round. Rating was different between the 2 reviewers for 9 articles. However agreement was found by consensus for all the articles.

The complete results are described in Table 1, where articles are first classified according to the population studied (TBI, ABI, or stroke), then classified according to the study design used (assessment of a predefined PF or identification of PFs among many variables). Hazard ratio (HR), odd ratio (OD), 95% confidence interval (CI), positive and negative likelihood ratio, sensitivity and specificity are reported when available. Complete RoB are reported in Table 2.

**Table 1:** Results

Population	Study type	Authors/publication year	Study design	*N*	Predictive factors	Principal results	Risk of bias analysis (QUIPS tool)
Traumatic brain injury (TBI)	Identification of PFs among many variables	Jenkins et al. [8]	- Retrospective observational study	79	- Demographic and admission variables (e.g., BMI), comorbidity, TBI mechanism, GCS) - Patient’s clinical variables (e.g., acute kidney failure, operating room trip, craniectomy, time with tracheostomy) - Stratification between decannulation prior to 90 days and after, and between variables present by hospital day 7 and prior to tracheostomy	PFs for lower likelihood of decannulation present on hospital day 7 prior to tracheostomy: - Diabetes (HR = 0.15; 95% CI (0.03–0.84); *p* = 0.03) - Acute kidney failure prior to day 7 (HR = 0.06; 95% CI (0.01–0.48); *p* = 0.01) - Craniotomy (HR = 0.25; 95% CI (0.06–1.02); *p* = 0 05) PFs for lower likelihood of decannulation present at any time during hospitalisation: - Reintubation (HR = 0.07; 95% CI (0.008–0.64); *p* = 0.02) - Aspiration (HR = 0.01; 95% CI (0.0–0.29); *p* = 0.01) - Craniotomy (HR = 0.00; 95% CI (0.0–0.39); *p* = 0.02) - Increased ventilator days after tracheostomy (HR = 0.74; 95% CI (0.572–0.947); *p* = 0.02) - Acute kidney injury (HR = 0.00; 95% CI (0.0–0.21); *p* = 0.01) PFs for higher likelihood of decannulation: - Increased age (HR = 1.12; 95% CI (1.008–1.214); *p* = 0.03) GCS at admission is not a PF for decannulation (*p* = 0.88)	Moderate
		Nowak et al. [14]	- Prospective observational study - No statistics available, only descriptive data	72	- Type of laryngeal and tracheal pathology found at endoscopy - Duration of intubation with endotracheal tube, tracheostomy tube, or both - Use of steroids and antibiotics during initial phases of management - Use and duration of a ventilator - Cognitive level at the time of decannulation - Cognitive function and airway outcome at 1-year follow-up examination	PFs for increased risk of mortality after decannulation: - RLAS level II and III PFs for longer time with tracheostomy: - Severe (laryngeal and tracheal) finding at endoscopy	High
		Klingbeil [17]	- Retrospective observational study - No statistics available, only descriptive data	44	Not clearly explained, descriptive article concerning retrospective study	PFs for no decannulation: - Injury severity - Tracheomalacia/tracheal stenosis PFs for failed decannulation: - Recurrent pneumonia - Palatal seizure - Laryngeal granulation - Tracheal stenosis	High
		Zanata et al. [15]	Prospective observational study	20	- Sex - GCS - Respiration (tube characteristic, cuff state) - Tracheal secretions (quality and quantity) - Phonation - Swallowing (clinical assessment, FOIS) - Coughing	PFs for decannulation: - Higher GCS - Tolerating tube capping - Low level of secretions - Absence of thick or yellowish secretions - Phonation - Absence of aspiration - Coughing	High
	Evaluation of predefined PFs	Ringrose et al. [39]	Retrospective observational study	51	PF: PSH vs no PSH	PSH associated with longer tracheostomy weaning	Moderate
Brain injury	Identification of PFs among many variables	Hakiki et al. [33] decannulation after severe ABI	- Retrospective observational study	351	Statistical analysis of 2 models (differences between the 2 models listed below are in italic) Model 1 - Aetiology - Presence of comorbidities - Pulmonary infection - Mechanical ventilation at admission - Tracheal alteration - Sepsis - FOIS at admission - *GCS at admission* - *CRS-r at admission* - Time between acute event and admission - LOS Model 2 - Aetiology - Presence of comorbidities - Pulmonary infections - Mechanical ventilation at admission - Tracheal alteration - Sepsis - FOIS at admission - *GCS* < *8 or GCS* > *8* - *Clinical state based on the CRS-r (i.e. E.MC, UWS, MCS)* - Time between acute event and admission - LOS	PFs for decannulation failure: - Sepsis (model 1: p = 0.001; HR, 0.526 and model 2: *p* < .001; HR, 0.448) - Tracheal alterations (model 1: *p* = 0.004; HR, 0.526; 95% CI (0.374–0.831) and model 2: *p* = 0.013; HR, 0.607; 95% CI (0.409–0.901)) - CRS-r at admission (model 1: *p* = 0.001; HR, 1.051; 95% CI (1.021–1.082)) - GCS < 8 at admission (model 2: *p* = 0.040; HR, 0.615; 95% CI (0.387 -0.978)) - E-MCS (*p* = 0.006; HR, 2.159; 95% CI (1.245 -3.745)) - MCS at admission (*p* < 0.001; HR, 2.617; 95% CI (1.584–4.324)) - Pulmonary infection (model 1: *p* < 0.001; HR, 0.302; 95% CI ( 0.199 -0.459) and model 2: *p* < .001; HR, 0.312; 95% CI (0.207–0.468)) PFs for delayed decannulation: - Pulmonary infection - Tracheal alteration - Sepsis PFs for shorter time to decannulation: - E-MCS and MCS vs UWS	Low
		Woo et al. (1989)	Prospective observational study	50	- Age - Type of injury - GCOS - Patient’s ability to swallow - Duration of tracheostomy - Presence of glottic sensation	PFs for decannulation: - Higher GCOS level - Injury type (TBI > anoxia or cerebral bleeding) - Younger age	High
		Heidler et al. (2018)	- Prospective multicentric observational study	470	- Sociodemographic and clinical data (relevant neurological and internal diseases) - Duration of mechanical ventilation, tracheostomy technique, and nutrition - CRS-r - Early Rehabilitation Barthel Index - Bogenhausener Dysphagia Score - Complications during the decannulation and rehabilitation periods (pneumonia, sepsis, laryngeal oedema, tracheal stenosis, tracheomalacia)	PFs for increased likelihood of decannulation: - Dilatational tracheostomy technique (OR 1.66; 95% CI (1.17–2.35); *p* = 0.005) - Oral diet (OR 3.80; 95% CI (2.00–7.21); *p* < 0.001) - Higher alertness (CRS-r) (OR 3.07 per 7.18 CRS-R points; 95% CI (2.47–3.83); *p* < 0.001) PFs for reduced likelihood of decannulation: - Age (older) (OR 0.68 per SD = 12.9 years; 95% CI (0.55–0.82); *p* < 0.001) - Male (OR 0.68; 95% CI (0.36–0.78); *p* = 0.001) - Longer mechanical ventilation (OR 0.57 per 33.2 days; 95% CI (0.45–0.70); *p* < 0.001) - CIPN/M (OR 0.66; 95% CI (0.44–1.00); *p* = 0.049) - Cardiac disease (especially chronic) (OR 0.5; 95% CI (0.32–0.78); *p* = 0.002) - Pneumonia (OR 0.32; 95% CI (0.19–0.51); *p* < 0.001) - Sepsis (OR 0.34; 95% CI (0.18–0.63); *p* = 0.001) - Other complications (OR 0.18; 95% CI (0.10–0.32); *p* < 0.001)	Low
		Mortensen et al. [30]	- Retrospective observational study	574	PFs chosen a priori by an expert panel depending on relevance and availability of hospital records: - Age (age groups: < 18, 18–40, 41–65, and > 65 years) - ABI diagnosis (stroke (ischaemic or haemorrhagic), TBI, SAH, encephalopathic brain injury, other injuries - Weeks from injury until admission to rehabilitation - EFA score	PFs for increased likelihood of successful decannulation: - Age < 18 years (generally younger age) (OR 4.23; 95% CI (2.36–7.52); *p* < 0.001) - EFA score of 61–100 with swallowing function at admission (OR 4.67; 95% CI (2.96–7.38); *p* < 0.001)	Low
		Lanini et al. [10]	Prospective observational study	194	- Sex - Age - GCS on admission - Cause of sABI (post-traumatic, post-anoxic, vascular, other) - Origin of patient (general, neurological, or cardiovascular ICU) - ICU LOS - Days from tracheostomy to rehabilitation unit - LOS in rehabilitation unit FBS was performed and tracheal lesions were treated if necessary	PFs for decannulation: - Younger age - Higher GCS on admission - Male - Shorter ICU LOS Reasons for decannulation failure: - Severe dysphagia with inability to manage oral secretions in 34 patients (47%) - Occurrence of acute events that prematurely stopped rehabilitation program in 22 patients (31%) - Late tracheostomy complications in 11 patients (15%) - Ineffective cough in 5 patients (7%) Decannulation failure is rarely caused by tracheal lesions	Moderate
		Mackiewicz-Nartowicz et al. [19]	Retrospective observational study	127	- Age - Sex - Aetiology (stroke, brain injury or cardiac arrest) - GCS in cases of trauma - Duration of tracheostomy - Tracheostomy complications - Concomitant diseases - Respiratory tract bacterial colonisation	PF for decannulation: - Age < 40 years PF for decannulation difficulties: - Longer time with tracheostomy	High
		Perin et al. (2017)	Retrospective observational study	45	- Age - Sex - BMI - GCS - ABI aetiology (stroke, trauma, cardiac arrest) - Date of pathological event - Gap between index event and first day of hospitalisation - LOS in neurorehabilitation - Comorbidities - Chest morphological alteration - Type of tracheostomy tube used (overall dimensions, cap, fenestration) - SpO_2_ - Presence and quantity of pulmonary secretions - MIP - MEP - Respiratory frequency and pattern - Cardiac frequency - Presence of spontaneous cough - Cough strength Blood gas analysis	PFs for decannulation: - TBI > stroke > anoxic - Presence of cough (OR 6.769; CI 95% (1.24–36.84) - Presence of spontaneous cough (OR 10; CI 95% (1.86–53.75) No effect of GCS No statistical effect of respiratory secretions, but these were considered principal reason for no decannulation	High
		Lui et al. [35]	Retrospective observational study	131	- Age - Sex - GCS - Pupil size on admission - Disease - Neurosurgical intervention - Timing of tracheostomy - Past medical history of pulmonary diseases cardiovascular diseases - Smoking status - Post-tracheostomy vocal cord status - Pneumonia with positive sputum culture within 1 month after tracheostomy	PFs for difficult decannulation group TW > 3 months: - Low GCS on admission - Presence of vocal cord palsy at 3 months - Presence of pneumonia within 1 month after tracheostomy	Moderate
		Mannini et al. [11]	Retrospective observational study	327	- Demographic data (age, sex) - Clinical data (aetiology, lesion localisation, time from onset, BMI, comorbidities) - FIM - FOIS - CRS-r - GCS - LCF - DRS - Vital support (nasogastric feeding tube, enteral nutrition, mechanical ventilation) - CIPN/M - Tracheal alterations The entire study used a data-driven approach to evaluate decannulation probability and timing based on ensemble learning models	PFs for increased likelihood of decannulation: - Younger age (OR 0.97; 95% CI (0.95–0.99); *p* < 0.001) - Female sex (OR 0.61; 95% CI (0.38–0.97); *p* < 0.05) - Higher CRS-r (OR 1.09; 95% CI (1.05–1.09); *p* < 0.001) - Higher GCS (OR 1.12; 95% CI (1.05–1.20); *p* < 0.001) - Higher FIM (OR 1.09; 95% CI (1.0–1.15); *p* < 0.001) - Higher LCF (OR 1.50; 95% CI (1.23–1.84); *p* < 0.001) - Lower DRS (OR 0.90; 95% CI (0.86–0.94); *p* < 0.001) - Lower DRS disability index (OR 0.58, 95% CI (0.47–0.72); *p* < 0.001) - Supratentorial lesion (OR 3.29; 95% CI (1.33–8.06); *p* < 0.05) - Absence of UWS (OR 0.25; 95% CI (0.15–0.43); *p* < 0.001) - Nasogastric tube (OR 4.65; 95% CI (1.82–11.76); *p* < 0.001) PFs for decreased likelihood of decannulation: - PEG (OR 0.20; 95% CI (0.07–0.55); *p* < 0.01) PFs for longer time to decannulation: - Older age - Higher BMI - Low LCF - Low FOIS - Low GCS - Low CRS-r - Low FIM - Presence of nasogastric feeding tube - Need for mechanical ventilation at admission - UWS - Haemorrhagic aetiology PFs for shorter time to decannulation: - Traumatic aetiology - Supratentorial lesions - PEG - E-MCS When the algorithm was used, decannulation was successfully predicted with an accuracy of 84.8% (AUC = 0.85) and timing was successfully estimated with a median absolute error of 25.7 days (IQR = 25.6)	Low
		Reverberi et al. [28]	Retrospective observational study	463	- Demographics - Date of onset and pathogenesis of brain lesion (anoxia, stroke, trauma, other causes) - Presence of vegetative status or minimal consciousness state - Saliva aspiration - Voluntary and reflex cough - FOIS	PFs for decannulation: - TBI > other causes > stroke > anoxic (OR 1.70; 95% CI (1.20–2.43); *p* = 0.003) - No vegetative state (OR 4.45; 95% CI (1.61–12.34); *p* = 0.004) - Efficient cough (voluntary + reflex > voluntary > reflex > none). (OR 1.56; 95% CI (1.14–2.15); *p* = 0.006) - Age tertile (OR 1.84; 95% CI (1.19–2.83); *p* = 0.006) - Saliva aspiration (OR 3.22; 95% CI (1.63–6.38); *p* = 0.001) Creation of DecaPreT tool: a small set of variables was included (age, pathogenesis of ABI, saliva aspiration, voluntary and reflex cough, and consciousness level). The ROC AUC of DecaPreT was 0.836	Moderate
	Evaluation of predefined PFs	Chan et al. [29]	Prospective observational study	32	Principal PF: - IPCF Secondary PFs: - GCS - Secretion volume	PFs for successful decannulation: - IPCF with 29 L/min threshold: positive predictive value, 78.3%; sensitivity, 85.7%; specificity, 54.5% (OR 1.12; 95% CI (1.02−1.23); *p* = 0.02) No effect of GCS and secretion volume	Moderate
		Enrichi et al. [32]	Prospective observational study	74	8 PFs based on Santus guidelines: - Voluntary cough - Cough reflex test - 72-h tube capping - Swallowing, instrument assessment using PAS - Blue-dye test - Number of tracheal inhalations - Instrument assessment of airway patency - SpO_2_ > 95% in ambient air - GCS ≥ 8 - Airway patency cluster (tube capping plus airway patency, instrument assessment) - Dysphagia cluster (blue-dye test plus swallowing, instrument assessment) - Clinical cluster (dysphagia cluster plus airway patency cluster)	PFs for decannulation: best values for sensitivity and specificity: - Tracheostomy tube capping (sensitivity, 80%; specificity, 100%; + LR NR; −LR 0.20 (0.08–0.48); *p* = 0.001; OR ∞) - Swallowing, instrument assessment using PAS ≤ 5 (sensitivity, 85%; specificity, 96.3%; + LR 22.95 (5.82−90.04); −LR 0.16 (0.05–0.44); *p* = 0.001; OR 122.90) - Number of tracheal suctions (≤ 2 every 8 h) (sensitivity, 70%; specificity, 92.6; + LR 9.45 (3.52−25.32); −LR 0.32 (0.16−0.63); *p* = 0.001; OR 26.94) - Blue-dye test (sensitivity, 65%; specificity, 85.1%; + LR 4.38 (2.14−8.97); −LR 0.41 (0.22−0.75); *p* = 0.001; OR 10.22) High specificity but low sensitivity: - Cough reflex test (sensitivity, 25%; specifcity, 98%; + LR 13.50 (1.67–108.58); −LR 0.76 (0.59–0.98); *p* = 0.001; OR 16.83) - SpO_2_ > 95% (sensitivity, 65%; specificity, 81.5%; + LR 3.51 (1.84–6.69); −LR; 0.43 (0.23–0.79); *p* = 0.001; OR 5.84) - GCS ≥ 8 (sensitivity, 55%; specificity, 92.6%; + LR 7.42 (2.67–20.65); −LR 0.48 (0.29–0.79); *p* = 0.001; OR 14.46) High sensitivity but low specificity: - Voluntary cough (sensitivity, 85%, specificity 31%; + LR 1.2 (0.95–1.60); −LR 0.47 (0.15–1.45); *p* = 0.23; OR 2.60) - Swallowing, instrument assessment using PAS = 1 (sensitivity, 85%; specificity 96.3; + LR 1.27 (0.98–1.66); −LR 0.45 (0.15–1.36); *p* = 0.15; OR 2.80) - Airway patency instrumental assessment (lumen ≥ 50%), (sensitivity, 100%; specificity, 30%; + LR NR; −LR 0.70; *p* = 0.001; OR ∞) , Airway patency cluster had high sensitivity (94.1%) and specificity (94.7%) (+ LR 17.88 (5.91–54.14), −LR 0.06 (0.01–0.42); *p* = 0.001; OR ∞) Dysphagia cluster had high sensitivity (94.4%) and specificity (81.8%) (+ LR 5.19 (2.93–9.2); −LR 0.07 (0.01–0.46); *p* = 0.001; OR 26.48). Decannulation clinical cluster had sensitivity of 100% and specificity of 82.5% (+ LR 5.75 (3.25–10.01); −LR NR; *p* = 0.01; OR ∞)	Low
		Leto et al. (2021)	Retrospective observational study	273	Use of revised DecaPreT tool (Reverberi, 2018) with new PFs: - Age at time of injury - CRS-r - ICU LOS	Probability of safe decannulation with an AUC of approximately 90% using new model	Low
		Bellon et al. (2022)	Retrospective observational case–control study	44	PF: DOC improvement assessed with CRS-r	PF for decannulation: - Patients with DOC improvements were 11-fold more likely to be decannulated, compared with patients who lacked such improvements (OR 11.28; 95% CI (1.96–123.08); *p* = 0.002)	Low
		Haikki et al. [33] polyneuropathy	Retrospective observational study	224	PF: CIPN/M	PF for delayed decannulation: - CIPN/M (presence of CIPN/M did not influence decannulation success)	Low
		Huang et al. [41]	Retrospective observational study	143	PF: early tracheostomy (day 1–10) vs late tracheostomy (after day 10) (infratentorial lesions only)	PFs for early decannulation: - Early tracheostomy (HR, 0.5; 95% CI 0.4–0.8; *p* = 0.003) - Tracheostomy caused by airway problems (HR, 0.42; 95% CI 0.28–0.63; *p* < 0.001) - No respiratory adverse events (HR, 1.78; 95% CI 1.22–2.60; *p* = 0.003)	Moderate
		Mitton et al. (2017)	Retrospective observational study	106	PF: lesion localisation: supratentorial vs infratentorial	PFs for unsuccessful decannulation: - Infratentorial lesion (no difference in time to decannulation between groups) - Excessive secretion load - Recurrent aspiration pneumonia PFs for delayed decannulation: - Oropharyngeal secretion management - Respiratory infection - Respiratory secretion management	Moderate
Stroke	Identification of PFs among many variables	Park and Lee [21]	Prospective observational study	101	VFSS (with FDS and PAS), PCF	PFs for successful decannulation: - Improvement in swallowing and cough - FDS total - FDS pharyngeal - PAS - K-MMSE - KMBI - PCF	Moderate
		Schneider et al. [23]	Prospective observational study	53	- Demographics - Comorbidities - NIHSS at admission - GCS - mRS - Brain imaging data (CT) - MRI - Lesion type (ICH, IS, SAH) - Lesion level (supratentorial or infratentorial) - Lesion location (left hemisphere, right hemisphere, or bilateral/central) - ACS - SET score on day of tracheostomy - Tracheostomy method - Experience of intensivists/surgeons performing tracheostomy - Tracheostomy-related procedural and early complications - mRS and Barthel index scores at 3 and 12 months - Patient care status (hospital, rehabilitation clinic, home, nursing home) - Mechanical ventilation - Complications with tracheostomy - Cannulation status - Stoma status	PFs for decannulation: - Younger age (HR 0.95; 95% CI (0.91–0.98); *p* = 0.002) - Absence of sepsis (HR 4.43; 95% CI (1.33–14.79); *p* = 0.008)	Low
		Reverberi et al. [28]	Retrospective observational study	463 (stroke subgroup *n* = 245)	PF analysis for stroke subgroup: - Demographics - Brain lesion date of onset - Pathogenesis (classified as anoxia, stroke, trauma, and other causes) - Vegetative status - Minimal consciousness state - Saliva aspiration - Voluntary and reflex cough - FOIS	PFs for successful decannulation: - Younger age (OR 1.94; 95% CI (1.28–2.93); (*p* = 0.002)) - No saliva aspiration (OR 3.29; 95% CI (1.75–6.20); *p* < 0.001)) - No vegetative status (OR 10.22; 95% CI (2.98–35.13); *p* < 0.001)) Efficient cough (OR 1.71; 95% CI (1.26–2.33) per each point increase in the coughing score (*p* = 0.001)) With these parameters, the ROC AUC of DecaPreT was 0.773	Moderate
		Küchler et al. [22]	Retrospective observational study	87	PFs: - Age - Sex - WFNS grade - Fisher grade - Presence of intracerebral or intraventricular haematoma - Acute hydrocephalus - Aneurysm location - Aneurysm obliteration (surgical vs endovascular) - Treatment-related complications - Decompressive craniectomy - Symptomatic CVS - Vasospasm-related infarction - Timing of tracheostomy - Pre-existing chronic lung disease - Pneumonia - mRS	PFs for delayed decannulation: - Older age (HR 2.11; 95% CI (1.22–3.64); *p* = 0.007)) - WFNS grade IV–V (HR 2.04; 95% CI (1.11–3.74); *p* = 0.022)) - Decompressive craniotomy (HR 2.16; 95% CI (1.23–3.77); *p* = 0.007)) - Occurrence of pneumonia (HR 2.00; 95% CI (1.18–3.42); *p* = 0.011)) PF for decannulation failure: - WFNS grade IV–V	Moderate
	Evaluation of predefined PFs	Gessler et al. [42]	Retrospective multicentric observational study	148	Principal PF: - Early (day 1–7) vs late (day 8–20) tracheostomy	PF for shorter decannulation: - Early tracheostomy (HR 0.5; 95% CI (0.31–0.79); *p* = 0.03)) PFs for delayed decannulation: - Age > 65 years (HR 4.02; 95% CI (1.95–8.26); *p* < 0.001)) - CVS (HR 1.65; 95% CI (1.03–2.63); *p* = 0.04))	Low

*ABI* acquired brain injury, *ACS* airway care score, *AUC* area under the curve, *BMI* body mass index, *CI* confidence interval, *CIPN/M* critical illness polyneuropathy/myopathy, *CRS-r* Coma Recovery Scale-revised, *CT* computed tomography, *CVS* cerebral vasospasm, *DOC* disorder of consciousness, *DRS* Disability Rating Scale, *EFA* early functional abilities, *E-MCS* emergence from minimal consciousness state, *FBS* flexible bronchoscopy, *FDS* Functional Dysphagia Scale, *FIM* Functional Independence Measurement, *FOIS* Food Oral Intake Scale, *GCOS* Glasgow Coma Outcome Scale, *GCS* Glasgow Coma Scale, *HR* hazard ratio, *ICH* intracerebral haemorrhage, *IPCF* induced peak cough flow, *ICU* intensive care unit, *IQR* interquartile range, *IS* ischaemic stroke, *KMBI* Korean Modified Barthel Index, *K-MMSE* Korean Mini Mental State Examination, *LCF* level of cognitive functioning, *LOS* length of stay,—*LR* negative likelihood ratio, + *LR* positive likelihood ratio, *MCS* minimal consciousness state, *MEP* maximum expiratory pressure, *MIP* maximum inspiratory pressure, *MRI* magnetic resonance imaging, *mRS* modified Rankin Scale, *NIHSS* National Institutes of Health Stroke Score, *NR* not reported; *OR* odd ratio, *PAS* Penetration–Aspiration Scale, *PCF* peak cough flow, *PEG* percutaneous endoscopic gastrostomy, *PF* predictive factor, *PSH* paroxysmal sympathetic hyperactivity, *QUIPS* Quality in Prognosis Study, *RLAS* Rancho Los Amigos Scale, *ROC* receiver operating characteristic, *sABI* severe acquired brain injury, *SAH* subarachnoid haemorrhage, *SET* stroke-related early tracheostomy, *TBI* traumatic brain injury, *TW* tracheostomy weaning, *UWS* unresponsive wakefulness state, *VFSS* video fluoroscopic swallowing studies, *WFNS* World Federation of Neurosurgical Surgeons

**Table 2:** Complete RoB: low RoB are written en Italics, moderate RoB are written in Underline, high RoB are written in Bold

	Study participation	Study attrition	Prognosis factor measurement	Outcome measurement	Study confounding	Statistical analysis and reporting	Overall rating bias
Jenkins et al. [8]	*Low*	Moderate	*Low*	Moderate	Moderate	*Low*	Moderate
Nowak et al. [14]	*Low*	Moderate	**High**	**High**	Moderate	*Low*	**High**
Klingbeil [17]	*Low*	Moderate	**High**	**High**	**High**	**High**	**High**
Zanata et al. [15]	Moderate	**High**	Moderate	**High**	**High**	*Low*	**High**
Ringrose et al. [39]	Moderate	Moderate	Moderate	Moderate	Moderate	Moderate	Moderate
Hakiki et al. [33] Decannulation after severe ABI	*Low*	*Low*	*Low*	*Low*	*Low*	*Low*	*Low*
Woo, Kelly, Krishner (1986)	Moderate	**High**	**High**	Moderate	Moderate	Moderate	**High**
Heidler et al. (2018)	*Low*	*Low*	*Low*	*Low*	Moderate	*Low*	*Low*
Mortensen et al. [30]	Moderate	Moderate	*Low*	Moderate	*Low*	*Low*	*Low*
Lanini et al. [10]	Moderate	*Low*	Moderate	Moderate	Moderate	Moderate	Moderate
Mackiewicz-Narkowicz et al. [19]	**High**	**High**	**High**	**High**	**High**	**High**	**High**
Perin et al. (2017)	Moderate	**High**	Moderate	Moderate	**High**	*Low*	**High**
Lui et al. [35]	*Low*	Moderate	Moderate	Moderate	Moderate	*Low*	Moderate
Mannini et al. [11]	*Low*	*Low*	*Low*	Moderate	*Low*	*Low*	*Low*
Reverberi et al. [28]	*Low*	*Low*	*Low*	Moderate	Moderate	*Low*	Moderate
Chan et al. [29]	*Low*	Low	Moderate	**High**	Moderate	*Low*	Moderate
Enrichi et al. [32]	*Low*	*Low*	*Low*	Moderate	Moderate	*Low*	*Low*
Leto et al. (2021) low	*Low*	Moderate	*Low*	*Low*	*Low*	*Low*	*Low*
Bellon et al. (2022)	*Low*	Moderate	*Low*	Moderate	Moderate	*Low*	*Low*
Hakiki et al. [33] polyneuropathy	*Low*	*Low*	*Low*	*Low*	*Low*	*Low*	*Low*
Huang et al. [41]	Moderate	Moderate	*Low*	Moderate	Moderate	*Low*	Moderate
Mitton et al. (2017)	*Low*	Moderate	Moderate	Moderate	*Low*	*Low*	Moderate
Park and Lee [21]	*Low*	**High**	Moderate	*Low*	Moderate	*Low*	Moderate
Schneider et al. [23]	*Low*	Moderate	*Low*	Moderate	*Low*	*Low*	Low
Küchler et al. [22]	*Low*	Moderate	*Low*	**High**	Moderate	*Low*	Moderate
Gessler et al. [42]	*Low*	*Low*	*Low*	**High**	*Low*	*Low*	*Low*

## Discussion

In this systematic review, we describe intrinsic and extrinsic PFs. Intrinsic PFs may be subdivided into PFs present before the ABI and PFs present only after the ABI. Only one extrinsic PF was identified; it was present only after the ABI.

### Intrinsic PFs Present Before ABI

#### Age

Younger age is reportedly a PF for decannulation in 10 studies, including 7 from the ABI subgroup [15–21] and 4 from the stroke subgroup [20–24]. Younger age is usually associated with better overall health and fewer comorbidities. Notably, there is no definitive cut-off for age, although Küchler et al. and Gessler et al. proposed that ages > 60 years or 65 years, respectively, should be used as cut-offs for delayed decannulation [23, 24].

In contrast, one study of patients with TBIs found that older age was a PF for decannulation [8]. The authors hypothesised that this observation was related to the small number of physicians who performed tracheostomies on younger patients. Moreover, the study cohort lacked geriatric patients, which may have been influenced by the demographic characteristics of patients with severe TBIs (i.e., frequently younger men: 55% are 0–44 years old, whereas 29% are ≥ 65 years old) [9]. Moreover, fewer tracheostomies are performed on older patients with TBIs because of the higher risk associated with the procedure in patients who are aged ≥ 65 years (72% mortality) [9]. Thus, older patients with tracheostomies may have better overall health or less severe TBIs.

#### Sex

The effect of sex was evaluated in 11 studies, and no effect was observed in 8 studies [8, 15, 20, 22, 23, 25, 27, 28]. Only one study found that male sex was a PF for decannulation [10], whereas two studies found that male sex was a PF for less frequent decannulation [16, 19]. However, selection bias may have been responsible for these observations because most patients were male in these studies (Heidler et al., 68%; Mannini et al., 64%; and Lanini et al., 63%).

#### Body Mass Index

Body mass index was evaluated in three studies; it was a PF for delayed decannulation in one study [11] but had no effect in two studies [25, 27]. A high body mass index can impair respiratory function and have a negative impact on tracheostomy weaning.

### Intrinsic PFs Present Only After ABI

#### Neurological Status

In total, 14 studies found that a higher neurological status was the most important PF for decannulation [8, 9, 16, 18, 20, 23, 24, 28–34]. This finding is not surprising because swallowing is strongly dependent on neurological control [35–37]. Central lesions can impair swallowing centres in the brainstem or modulators in the cerebral hemispheres [12]. Moreover, diffuse lesions can severely impair alertness; therefore, dysphagia may occur regardless of whether swallowing centres are intact [13].

There is no consensus regarding the method for measurement of neurological status. Therefore, various scales were observed among studies in this review (see supplemental file 1 for a complete description of all the scales reported in this review).

In the TBI subgroup, Nowak et al. used the Rancho Los Amigos Scale (RLAS), a cognitive behavioural scale designed to evaluate patients with ABIs who are recovering from comas [14]. The RLAS used in Nowak et al*.* is the first iteration and comprises 8 levels: I No response to deep pain stimulus; II Generalized response to deep pain stimulus; III Localized response to deep pain stimulus; IV Confused, agitated; V Confused, inappropriate, not agitated; VI Confused, appropriate; VII Automatic, appropriate; VII Purposeful, appropriate. Jenkins et al. and Zanata et al. used the Glasgow Coma Scale (GCS) at admission [8] and at the latest assessment [15], respectively. The GCS is probably the most widely used behavioral scale used to assess the severity of TBI at the acute phase. It is composed of 3 subscale scores (eye-opening, verbal and motor). The total score is ranging from 3 to 15 (scores of 3–8 indicating a severe injury, 9–12 a moderate injury, and 13–15 a mild injury) [16]. Klingbeil did not report any scale [17]. In one study, craniotomy was a PF for a lower likelihood of decannulation [8]. Craniotomy may be associated with a worse neurological status because this surgical procedure is performed on patients with intracranial hypertension [18]. Conversely, Jenkins et al. found that GCS score at admission was not a PF for decannulation, presumably because of the many confounders involved at that time, such as the use of sedation [8].

In the ABI subgroup, the following scales were used: Coma Recovery Scale-revised (CRS-r) (at admission or later) in five studies [16, 19, 31, 33, 41], GCS (at admission or later; cut-off, GCS < 8) in eight studies [9, 15, 18, 19, 27, 28, 31, 42], and level of cognitive functioning (LCF) in one study [11]. Moreover, functional scales were used to assess disabilities related to the severity of ABIs. These included the Glasgow Coma Outcome Scale (GCOS), the Functional Independence Measurement (FIM), the Disability Rating Scale (DRS), and the Early Functional Abilities (EFA) scale. Better functional status was a PF for decannulation in three studies [17, 19, 21]. Improvements in the Disorders of Consciousness (DoC) or Emergence from Minimal Consciousness State (E-MCS) were also PFs for decreasing the time until decannulation [41]. However, three studies did not find that the GCS was a PF for decannulation [15, 27, 42]. This observation may be explained by the characteristics of each population: all patients with TBIs had a GCS score of < 6 at admission [19], or the correlation between a higher GCS and decannulation showed a tendency that failed to reach statistical significance [27, 42]. Importantly, the GCS was designed for patients with TBIs and may lack precision when applied to patients with ABIs (e.g. stroke). Therefore its use should be reserved for TBI patients. [16]. Conversely, the CRS-r was more reliable for predicting decannulation failure, difficulties, or delayed decannulation. The CRS-r is a standardized and validated assessment measure of the neurobehavioral status of brain-injured patients. It is also used to detect subtle improvements in disorders of consciousness [20]. It is composed of 6 subscales for assessment of oromotor, communication, auditory, visual, motor and alertness process. It is organized in 29 items. Patients can be rated between 0 (minimum score corresponding to an Unresponsible Wakefullness Syndrome (UWS) and 23 (maximum score, corresponding to a normal and complete conscious state) [20].

In the stroke subgroup, the results were less clear. Some of the indicators used were more relevant for the consequences or severity of a stroke (e.g., the Korean Mini Mental State Examination (K-MMSE) score, the Korean Modified Barthel Index (K-MBI), decompressive craniotomy, the World Federation of Neurosurgical Societies (WFNS) scale, and the presence of cerebral vasospasm). The PFs associated with successful decannulation included a higher K-MMSE score, a higher K-MBI, and the absence of vegetative status [21]. PFs associated with delayed decannulation included decompressive craniotomy and cerebral vasospasm [23, 24]. One study found that a WFNS grade of IV–V was a PF for decannulation failure [22]. The GCS was only used in one of these studies [23] and was not a PF: the GCS score was higher in the decannulated group, but the difference was not statistically significant.

#### Lesion Localisation

Two studies [19] evaluated the impact of lesion localisation; they found that the presence of infratentorial lesions was a PF for decannulation failure, presumably because neurological control of swallowing is principally supported by brainstem structures [24]. Infratentorial lesions may damage these structures and impair swallowing.

#### Type of Lesion

Lesion type was a PF for decannulation failure in four studies [19–21, 27]. Patients who had lesions with traumatic rather than vascular causes were more likely to experience successful decannulation. Patients with anoxic lesions were least likely to experience successful decannulation, presumably because of the poor overall prognosis for anoxic patients [25].

#### Coughing

A strong cough was a positive PF in five studies [8, 18, 20, 27, 42]. Coughing protect the respiratory airway. When cough is impaired, patients experience a greater risk of pulmonary infections. Coughing clears the airway, but the vocal cords and inspiratory/expiratory muscles must exhibit sufficient function to maintain a strong cough [26]. An ABI can impair coughing [27] and increase the risk of pulmonary infections.

In the TBI subgroup, one study highlighted parameters associated with respiratory function that are assessed during tracheostomy weaning, including an effective cough, phonation, and the quality and quantity of tracheal secretions [15].

In the ABI subgroup, a strong cough was a PF for successful decannulation in three studies [20, 27, 42]. One study showed that a weak cough was a reason for decannulation failure [10]. Additionally, Reverberi et al. found that the combined presence of a voluntary cough and a reflex cough was a more reliable PF than the presence of a voluntary cough alone, followed by the presence of a reflex cough alone; the absence of coughing was a poor PF for decannulation [28]. The strength of a voluntary cough can be measured using a peak cough flow (PCF); a cut-off of 160 L/min has been proposed [7, 11]. However, this value is most relevant for patients with neuromuscular diseases, rather than patients with ABIs [2]. Moreover, reduced levels of alertness may hinder the assessment of a voluntary cough in patients with ABIs. Consequently, Chan et al. proposed the use of induced peak cough flow (IPCF) to assess cough strength [42] where the cough reflex is induced by touching the tracheal mucosa with a suction catheter through a tracheostomy tube; cough strength is recorded using an electronic peak flow meter. Chan et al. proposed a rather low peak flow rate threshold of 29 L/min [29].

In the stroke subgroup, two studies [20, 34] found that an effective cough was a PF for successful decannulation. To evaluate coughing, Park and Lee used the PCF measurement. However, only 31 of 101 patients were able to complete the measurement because of cognitive impairments [21]. This is similar with Chan et al. who proposed measuring IPCF because the assessment of PCF requires voluntary control [29]. Reverberi et al. emphasised the importance of an effective cough [20] for decannulation.

#### Swallowing

Effective swallowing was a positive PF in five studies [8, 18, 20, 27, 42]. Similar to an effective cough, effective swallowing protects the airway, whereas an impaired swallowing may increase the risk of pulmonary infections and thus decannulation failure.

In the TBI subgroup, one study highlighted parameters associated with swallowing function that are assessed during tracheostomy weaning: tube capping, safe swallowing (with no sign of aspiration), phonation, and the quality and quantity of tracheal secretions [15].

In the ABI subgroup, three studies found that effective swallowing was a PF for decannulation [9, 17, 18]. Lanini et al. reported that severe dysphagia and an inability to manage oral secretions resulted in decannulation failure in 34 patients (47%) [10]. Mortensen et al. assessed patients with different EFA scores combined with the swallowing item (i.e., 20–40 plus no swallowing, 20–40 plus swallowing, 41–60 plus no swallowing, 41–60 plus swallowing, 61–100 plus no swallowing, or 61–100 plus swallowing) [30]. They found that an EFA score of 61–100 combined with effective swallowing was a PF for successful decannulation. The EFA score is used to assess patients with severe disabilities, usually within the first 72 h after admission. It comprises 20 items in 4 categories (autonomic, oro-facial, sensorimotor and cognitive functions/abilities). More specifically the 6th and 7th items are referring respectively to the swallowing function and the tongue movements and chewing. The EFA total score is ranging from 20 to 100, higher scores indicating better abilities. This score is a reliable tool to predict outcomes [31].

Enrichi et al. created a ‘dysphagia cluster’ comprising fibreoptic evaluation of swallowing using the Penetration–Aspiration Scale (PAS) score and a cut-off of ≤ 5 (i.e., swallowing difficulties with penetration but no aspiration), in combination with the blue-dye test (the positive criterion was the absence of a blue trace) [32]. Fibreoptic evaluation is used to assess penetration/aspiration and pharyngeal residues. The blue-dye test is used to assess aspiration alone. The two tests are complementary. The ‘dysphagia cluster’ exhibited 94.4% sensitivity and 81.8% specificity. Moreover Enrichi et al. also found that mild dysphagia, characterised by pharyngeal residues or penetration (PAS score, 2–5), was not a negative PF for decannulation [32]. Consequently, patients with mild dysphagia may benefit from decannulation. However, severe dysphagia is a negative PF for decannulation. Furthermore Enrichi et al. used clusters of clinical parameters to evaluate PFs for decannulation, including airway patency (tube capping and instrument assessment) and dysphagia (instrument assessment and the blue-dye test). Both clusters exhibited high specificity and sensitivity. The authors then combined these clusters (airway patency and dysphagia) to created the “clinical cluster”, that had a sensitivity of 100% and a specificity of 82.5% [32]. This cluster was used to assess severe swallowing disorders and impaired airway patency, which can cause major problems during tracheostomy weaning.

In the stroke subgroup, two studies [20, 34] found that an effective swallowing was a PF for successful decannulation. Park and Lee evaluated improvements in swallowing using video fluoroscopic swallowing studies (VFSS), in combination with the Functional Dysphagia Scale (FDS) and the PAS [21]. Reverberi et al. emphasised the importance of the lack of saliva aspiration for decannulation, which was assessed using the blue-dye test [28].

#### Tracheal Lesions

Three studies, two involving patients with TBIs [29, 30] and one involving patients with ABIs [33], found that tracheal lesions were negative PFs for decannulation. Lesions such as granulomas, oedemas, and tracheomalacias may decrease airway patency [34], thereby hindering tracheostomy weaning (especially tube capping). However, one study [10] found that the presence of tracheal lesions rarely causes decannulation failure. In that study, only 11 of 194 patients could not be decannulated because of tracheal lesions that did not respond to treatment. However, tracheal lesions were present in 82% of the included patients. Therefore, lesions are very common, but the problem can usually be addressed by using a smaller cannula [2]. Enrichi et al. proposed an evaluation of airway patency based on tube capping and instrumental assessment [32]. However, they did not provide recommendations for the treatment of tracheal lesions or improvement of airway patency. One study found that vocal cord palsy was a PF for difficulties during decannulation [35]. Vocal cord palsy may be associated with aspiration, a weak cough, or decreased airway patency, leading to difficulties during tracheostomy weaning [12].

#### Pulmonary Infections

Five studies found that pulmonary infections were negative PFs for decannulation [16, 23, 28, 31]. In the ABI subgroup, pulmonary infections were PFs for decannulation failure, delayed decannulation, reduced likelihood of decannulation, and difficulties during decannulation in four studies [16, 28, 31]. Pulmonary infections can severely impair respiratory function, hindering tracheostomy weaning. Additionally, pulmonary infections may result from swallowing disorders [12]. Patients with severe swallowing disorders are more likely to develop pulmonary infections that make them ineligible for tracheostomy weaning and decannulation. Infection treatment alone may not ensure that these patients can be safely decannulated, and instrument-based swallowing evaluations may be necessary. In the stroke subgroup, one study found that pneumonia was a PF for delayed decannulation [22]. Another study found that the absence of sepsis was a PF for decannulation [23], whereas pneumonia was not. Although the difference was not statistically significant, there were more cases of pneumonia in the non-decannulated group (6 cases, *n* = 34) than in the decannulated group (1 case, *n* = 19). In total, there were only 7 cases of pneumonia in 53 patients. In the study by Küchler et al., 64 of 87 patients had pneumonia, including all patients in the non-decannulated group [22]. One explanation for this difference is that all patients in the first study had poor-grade subarachnoid haemorrhages. Indeed, Schneider et al. studied patients with severe stroke (median National Institutes of Health Stroke score, 32; interquartile range, 22.5–32) [23]. Nevertheless, the reason for the discrepancy between the two studies is unclear.

#### CIPN/M

Two studies found that CIPN/M was a PF for decreased likelihood of decannulation and for delayed decannulation [16]. Critical illness polyneuropathy (CIP), critical illness myopathy (CIM), or critical illness neuromyopathy (CINM) are different classifications of ICU-aquired weakness (ICU-AW). This condition is frequent in patients who survived to ICU stay with a prevalence of ~ 46%; 95% CI 43–49% [36]. It is ‘a clinically detected weakness in critically ill patients in whom there is no plausible aetiology other than critical illness’. It is also considered to be an independent factor for worse outcomes [37]. The presence of these critical illnesses can impair coughing, swallowing, and respiratory function, leading to difficulties during tracheostomy weaning. Nevertheless, CIPN/M is usually reversible. Therefore, Haikki et al. found that CIPN/M was a PF for delayed decannulation but not for decannulation failure [38].

#### Paroxysmal Sympathetic Hyperactivity (PSH)

In the TBI subgroup, one study found that PSH was a PF for delayed decannulation but was not associated with decannulation failure [39]. PSH frequently occurs in patients with TBIs [39]. These parasympathetic disorders (e.g., tachypnoea and tachycardia) can lead to difficulties during tracheostomy weaning [40]. However, in patients with TBIs, PSH usually subsides over time [40]. Therefore, PSH is a PF for delayed tracheostomy weaning but not for decannulation failure.

### Extrinsic PF Present Only After ABI

#### Early Tracheostomies

Two studies found that an early tracheostomy is a positive PF for decannulation [24]. Early tracheostomies may promote rehabilitation and allow earlier consideration of decannulation. In the ABI subgroup, Huang et al. [41] found that an early tracheostomy (day 1 to 10) was a PF for early decannulation, compared with late tracheostomy (after day 10). Huang et al. only studied patients with infratentorial lesions, but those lesions had various aetiologies including gliomas, meningiomas, neurilemmomas, and vascular malformations [41]. In the stroke subgroup, Gessler et al. also found that an early tracheostomy was a PF for early decannulation [42].

#### Mechanical Ventilation

Six studies have tested mechanical ventilation as a PF. Results are unclear, probably because of the heterogeneity of the outcomes studies. Three studies found that the need for mechanical ventilation and a longer mechanical ventilation duration are PFs for a lower likelihood of decannulation or a delayed decannulation (Jenkins 2020; Heidler 2018, Manini 2021). Conversely, 3 studies did not find any relation between mechanical ventilation and decannulation. However in Nowak et al. mechanical ventilation duration was very short, ranging from 2 to 7 days. In Haikki et al*.* and in Schneider et al*.* it is only the presence of mechanical ventilation at admission that was studied, data on mechanical ventilation duration are not available. Therefore it is difficult to conclude on the impact on mechanical ventilation and mechanical ventilation duration on decannulation in this population. More studies are needed to answer this question.

### Study Limitations

One of the main limitations of this review was that most included studies involved the identification of PFs among many potential variables. The tested variables often differed among studies, and some variables were only tested in one study. Additionally, the most frequently reported PF was neurological status; this was also the most frequently tested and (arguably) most anticipated PF. The frequency and expectation aspects are important sources of potential bias. Furthermore, many of these studies were retrospective, and only available data could be tested. Importantly, the data recorded may differ among studies because of distinct clinical environments and local procedures. Few studies focused on specific variables that were considered potential PFs a priori, and only one PF (early vs late tracheostomy) was tested twice [24, 53]. Additionally, only 10 of the 26 studies included in this review had a low RoB; most included studies were retrospective, and with many confounding factors. The outcomes considerably varied among studies; they included decannulation failure, delayed decannulation, likelihood of decannulation, and decannulation success. Importantly, these outcomes are not equivalent: a delayed decannulation may also be successful. Conversely, early decannulation may be followed by recannulation, and an increased likelihood of decannulation does not necessarily increase the likelihood of successful decannulation. Finally, tracheostomy weaning and decannulation protocols frequently differed among studies. For example, some studies used instrumental assessments and evaluated PFs that could not be compared with other studies. Similarly, decisions to decannulate were based on clinical factors that may have varied among studies. Confounding factors may also have arisen because of the different methods used.

Although it was largely based on retrospective observational studies that focused on different populations (i.e., patients with TBIs, ABIs, and stroke), this systematic review of the literature identified several PFs for successful decannulation in patients with brain injuries. These PFs included high neurological status, TBIs rather than stroke or anoxic brain lesions, younger age, effective swallowing, an effective cough, and the absence of pulmonary infections. Less frequently reported PFs that may be more applicable to aetiological subgroups included early tracheostomy, supratentorial lesions, the absence of CIPN/M, and the absence of tracheal lesions. These PFs may be used by clinicians before decannulation and during tracheostomy weaning. However, prospective studies with more reliable methodologies are needed to validate these PFs and identify others.

### Supplementary Information

Below is the link to the electronic supplementary material.

Supplementary file1 (DOCX 1278 KB)

## References

[1] Durbin CGJ (2010). Tracheostomy: why, when, and how?. Respir Care.

[2] Singh RK, Saran S, Baronia AK (2017). The practice of tracheostomy decannulation—a systematic review. J Intensive Care.

[3] Santus P, Gramegna A, Radovanovic D, Raccanelli R, Valenti V, Rabbiosi D (2014). A systematic review on tracheostomy decannulation: a proposal of a quantitative semiquantitative clinical score. BMC Pulm Med.

[4] de Medeiros GC, Sassi FC, Lirani-Silva C, de Andrade CRF (2019). Critérios para decanulação da traqueostomia: revisão de literatura. CoDAS.

[5] Bösel J (2014). Tracheostomy in stroke patients. Curr Treat Options Neurol.

[6] Grooten WJA, Tseli E, Äng BO, Boersma K, Stålnacke BM, Gerdle B (2019). Elaborating on the assessment of the risk of bias in prognostic studies in pain rehabilitation using QUIPS—aspects of interrater agreement. Diagn Progn Res.

[7] Hayden JA, Van Der Windt DA, Cartwright JL, Côté P, Bombardier C (2013). Assessing bias in studies of prognostic factors. Ann Intern Med.

[8] Jenkins R, Badjatia N, Haac B, Van Besien R, Biedlingmaier JF, Stein DM (2020). Factors associated with tracheostomy decannulation in patients with severe traumatic brain injury. Brain Inj.

[9] Majdan M, Plancikova D, Brazinova A, Rusnak M, Nieboer D, Feigin V (2016). Epidemiology of traumatic brain injuries in Europe: a cross-sectional analysis. Lancet Public Health.

[10] Lanini B, Binazzi B, Romagnoli I, Chellini E, Pianigiani L, Tofani A (2021). Tracheostomy decannulation in severe acquired brain injury patients: the role of flexible bronchoscopy. Pulmonology.

[11] Mannini A, Hakiki B, Liuzzi P, Campagnini S, Romoli A, Draghi F (2021). Data-driven prediction of decannulation probability and timing in patients with severe acquired brain injury. Comput Methods Progr Biomed.

[12] Dziewas R, Allescher HD, Aroyo I, Bartolome G, Beilenhoff U, Bohlender J (2021). Diagnosis and treatment of neurogenic dysphagia – S1 guideline of the German Society of Neurology. Neurol Res Pract.

[13] Bremare A, Rapin A, Veber B, Beuret-Blanquart F, Verin E (2016). Swallowing disorders in severe brain injury in the arousal phase. Dysphagia.

[14] Nowak P, Cohn AM, Guidice MA (1987). Airway complications in patients with closed-head injuries. Am J Otolaryngol.

[15] de Zanata IL, Santos RS, Hirata GC (2014). Tracheal decannulation protocol in patients affected by traumatic brain injury. Int Arch Otorhinolaryngol.

[16] Bodien YG, Barra A, Temkin NR, Barber J, Foreman B, Vassar M (2021). Diagnosing level of consciousness: the limits of the Glasgow coma scale total score. J Neurotrauma.

[17] Klingbeil GEG (1988). Airway problems in patients with traumatic brain injury. Arch Phys Med Rehabil.

[18] Alvis-Miranda H, Castellar-Leones SM, Moscote-Salazar LR (2013). Decompressive craniectomy and traumatic brain injury: a review. Bull Emerg Trauma.

[19] Mackiewicz-Nartowicz H, Mackiewicz-Milewsk M, Lach S, Szymańska-Skrzypek A, Owczarek A, Sinkiewicz A (2008). Decannulation factors in patients after serious brain injuries. Adv Palliat Med.

[20] Lucca LF, Lofaro D, Pignolo L, Leto E, Ursino M, Cortese MD (2019). Outcome prediction in disorders of consciousness: the role of coma recovery scale revised. BMC Neurol.

[21] Park MK, Lee SJ (2018). Changes in swallowing and cough functions among stroke patients before and after tracheostomy decannulation. Dysphagia.

[22] Küchler J, Wojak JF, Smith E, Brocke J, Abusamha A, Tronnier VM (2019). Management of tracheostomized patients after poor grade subarachnoid hemorrhage: disease related and pulmonary risk factors for failed and delayed decannulation. Clin Neurol Neurosurg.

[23] Schneider H, Hertel F, Kuhn M, Ragaller M, Gottschlich B, Trabitzsch A (2017). Decannulation and functional outcome after tracheostomy in patients with severe stroke (DECAST): a prospective observational study. Neurocrit Care.

[24] Dodds WJ (1989). The physiology of swallowing. Dysphagia.

[25] Cullen NK, Crescini C, Bayley MT (2009). Rehabilitation outcomes after anoxic brain injury: a case-controlled comparison with traumatic brain injury. PM&R.

[26] McCool FD (2006). Global physiology and pathophysiology of cough. Chest.

[27] Ward K, Seymour J, Steier J, Jolley CJ, Polkey MI, Kalra L (2010). Acute ischaemic hemispheric stroke is associated with impairment of reflex in addition to voluntary cough. Eur Respir J.

[28] Reverberi C, Lombardi F, Lusuardi M, Pratesi A, Di Bari M (2019). Development of the decannulation prediction tool in patients with dysphagia after acquired brain injury. J Am Med Dir Assoc.

[29] Chan LYY, Jones AYM, Chung RCK, Hung KN (2010). Peak flow rate during induced cough: a predictor of successful decannulation of a tracheotomy tube in neurosurgical patients. Am J Crit Care.

[30] Mortensen J, Kjeldsen SS, Honoré H, Pedersen AR (2020). Using routinely gathered clinical data to develop a prognostic online tool for decannulation in subjects with acquired brain injury. Respir Care.

[31] Hankemeier A, Rollnik JD (2015). The Early Functional Abilities (EFA) scale to assess neurological and neurosurgical early rehabilitation patients. BMC Neurol.

[32] Enrichi C, Battel I, Zanetti C, Koch I, Ventura L, Palmer K (2017). Clinical criteria for tracheostomy decannulation in subjects with acquired brain injury. Respir Care.

[33] Hakiki B, Draghi F, Pancani S, Portaccio E, Grippo A, Binazzi B (2020). Decannulation after a severe acquired brain injury. Arch Phys Med Rehabil.

[34] Meenan K, Bhatnagar K, Guardiani E (2021). Intubation-related laryngeal pathology precluding tracheostomy decannulation: incidence and associated risk factors. Ann Otol Rhinol Laryngol.

[35] Lui HCH, He Z, Zhuang TF, Ng CF, Wong GKC (2021). Tracheostomy decannulation outcomes in 131 consecutive neurosurgical patients. British J Neurosurg.

[36] Stevens RD, Dowdy DW, Michaels RK, Mendez-Tellez PA, Pronovost PJ, Needham DM (2007). Neuromuscular dysfunction acquired in critical illness: a systematic review. Intensive Care Med.

[37] Appleton R, Kinsella J (2012). Intensive care unit-acquired weakness. Continuing Educ Anaesth Crit Care Pain.

[38] Hakiki B, Draghi F, Scarpino M, Portaccio E, Romoli A, Mannini A (2020). Critical illness polyneuromyopathy: functional impact after severe acquired brain injuries. Acta Neurol Scand.

[39] Ringrose H, Brown M, Walton K, Sivan M (2018). Association between paroxysmal sympathetic hyperactivity and tracheostomy weaning in traumatic brain injury. NRE.

[40] Meyfroidt G, Baguley IJ, Menon DK (2017). Paroxysmal sympathetic hyperactivity: the storm after acute brain injury. Lancet Neurol.

[41] Huang HW, Zhang GB, Xu M, Chen GQ, Zhang XK, Zhang JT (2021). The impact of tracheostomy timing on clinical outcomes and adverse events in intubated patients with infratentorial lesions: early versus late tracheostomy. Neurosurg Rev.

[42] Gessler F, Mutlak H, Lamb S, Hartwich M, Adelmann M, Platz J (2015). The impact of tracheostomy timing on clinical outcome and adverse events in poor-grade subarachnoid hemorrhage. Crit Care Med.

