# Supplementary material for: Predictive Factors for Successful Decannulation in Patients with Tracheostomies and Brain Injuries: A Systematic Review
Source: Dysphagia. 2024 Jan 8;39(4):552–72. doi: 10.1007/s00455-023-10646-2 (PMC11239766; doi:10.1007/s00455-023-10646-2)
Supplement: Supplementary file 1 — Supplementary file1 (DOCX 1278 KB) [file 455_2023_10646_MOESM1_ESM.docx]

**Supplemental files 1: Scales found in the review (by order of citation in table 1)**

**Glasgow coma scale (GCS):**

***Best eye response (4)***

1. No eye opening
2. Eye opening to pain
3. Eye opening to sound
4. Eyes open spontaneously

***Best verbal response (5)***

1. No verbal response
2. Incomprehensible sounds
3. Inappropriate words
4. Confused
5. Orientated

***Best motor response (6)***

1. No motor response.
2. Abnormal extension to pain
3. Abnormal flexion to pain
4. Withdrawal from pain
5. Localizing pain
6. Obeys commands

Jain S, Iverson LM. Glasgow Coma Scale. [Updated 2022 Jun 21]. In: StatPearls [Internet]. Treasure Island (FL): StatPearls Publishing; 2023 Jan-. Available from: <https://www.ncbi.nlm.nih.gov/books/NBK513298/>

**Rancho Los Amigos Scale (RLAS) :**

**Level I: No Response: Total Assistance**

- No response to external stimuli

**Level II: Generalized Response: Total Assistance**

- Responds inconsistently and non-purposefully to external stimuli
- Responses are often the same regardless of the stimulus

**Level III: Localized Response:** **Total Assistance**

- Responds inconsistently and specifically to external stimuli
- Responses are directly related to the stimulus, for example, patient withdraws or vocalizes to painful stimuli
- Responds more to familiar people (friends and family) versus strangers

**Level IV: Confused/Agitated: Maximal Assistance**

- The individual is in a hyperactive state with bizarre and non-purposeful behavior
- Demonstrates agitated behavior that originates more from internal confusion than the external environment
- Absent short-term memory

**Level V: Confused, Inappropriate Non-Agitated:** **Maximal Assistance**

- Shows increase in consistency with following and responding to simple commands
- Responses are non-purposeful and random to more complex commands
- Behavior and verbalization is often inappropriate, and individual appears confused and often confabulates
- If action or tasks is demonstrated individual can perform but does not initiate tasks on own
- Memory is severely impaired and learning new information is difficult
- Different from level IV in that individual does not demonstrate agitation to internal stimuli. However, they can show agitation to unpleasant external stimuli.

**Level VI: Confused, Appropriate: Moderate Assistance**

- Able to follow simple commands consistently
- Able to retain learning for familiar tasks they performed pre-injury (brushing teeth, washing face) however unable to retain learning for new tasks
- Demonstrates increased awareness of self, situation, and environment but unaware of specific impairments and safety concerns
- Responses may be incorrect secondary to memory impairments but appropriate to the situation

**Level VII: Automatic, Appropriate: Minimal Assistance for Daily Living Skills**

- Oriented in familiar settings
- Able to perform daily routine automatically with minimal to absent confusion
- Demonstrates carry over for new tasks and learning in addition to familiar tasks
- Superficially aware of one’s diagnosis but unaware of specific impairments
- Continues to demonstrate lack of insight, decreased judgment and safety awareness
- Beginning to show interest in social and recreational activities in structured settings
- Requires at least minimal supervision for learning and safety purposes.

**Level VIII: Purposeful, Appropriate: Stand By Assistance**

- Consistently oriented to person, place and time
- Independently carries out familiar tasks in a non-distracting environment
- Beginning to show awareness of specific impairments and how they interfere with tasks, however, requires standing by assistance to compensate
- Able to use assistive memory devices to recall daily schedule
- Acknowledges other’s emotional states and requires only minimal assistance to respond appropriately
- Demonstrates improvement of memory and ability to consolidate the past and future events
- Often depressed, irritable and with low frustration threshold

**Level IX: Purposeful, Appropriate: Stand By Assistance on Request**

- Able to shift between different tasks and complete them independently
- Aware of and acknowledges impairments when they interfere with tasks and able to use compensatory strategies to cope
- Unable to independently anticipate obstacles that may arise secondary to impairment
- With assistance able to think about consequences of actions and decisions
- Acknowledges the emotional needs of others with stand by-assistance.
- Continues to demonstrate depression and low frustration threshold

**Level X: Purposeful, Appropriate: Modified Independent**

- Able to multitask in many different environments with extra time or devices to assist
- Able to create own methods and tools for memory retention
- Independently anticipates obstacles that may occur as a result of impairments and take corrective actions
- Able to independently make decisions and act appropriately but may require more time or compensatory strategies
- Demonstrate intermittent periods of depression and low frustration threshold when under stress
- Able to appropriately interact with others in social situations

Lin K, Wroten M. Ranchos Los Amigos. [Updated 2022 Aug 22]. In: StatPearls [Internet]. Treasure Island (FL): StatPearls Publishing; 2023 Jan-. Available from: <https://www.ncbi.nlm.nih.gov/books/NBK448151/>

**Functional Oral Intake Scale (FOIS) :**

TUBE DEPENDENT (levels 1-3)

1 No oral intake

2 Tube dependent with minimal/inconsistent oral intake

3 Tube supplements with consistent oral intake

TOTAL ORAL INTAKE (levels 4-7)

4 Total oral intake of a single consistency

5 Total oral intake of multiple consistencies requiring special

preparation

6 Total oral intake with no special preparation, but must avoid

specific foods or liquid items

7 Total oral intake with no restrictions

Crary MA, Carnaby-Mann GD, Groher ME. Initial psychometric assessment of a functional oral intake scale for dysphagia in stroke patients. Arch Phys Med Rehabil 2005;86:1516-1520.

**Coma Recovery Scale revised (CRS-r)**

Auditory function scale

4 – Consistent Movement to Command

3 – Reproducible Movement to Command

2 – Localization to Sound

1 – Auditory Startle

0 – None

Visual function scale

5 – Object Recognition

4 – Object localization: Reaching

3 – Visual Pursuit

2 – Fixation

1 – Visual Startle

0 – None

Motor function scale

6 – Functional Object Use

5 – Automatic Motor Response

4 – Object Manipulation

3 – Localisation to Noxious Stimulation

2 – Flexion Withdrawal

1 – Abnormal Posturing

0 – None

Oromotor / Verbal function scale

3 – Intelligible Verbalization

2 – Vocalization/Oral Movement

1 – Oral Reflexive Movement

0 – None

Communication scale

2 – Functional: Accurate

1 – Non-functional: Intentional

0 – None

Arousal scale

3 – Attention

2 – Eye Opening w/o Stimulation

1 – Eye Opening with Stimulation

0 – Unarousable

Giacino JT. ÉCHELLE DE RÉCUPÉRATION DU COMA VERSION REVUE FRANÇAISE ©2008. :15.

**Glasgow Coma Outcome Scale (GCOS) :**

1. Dead: As a direct result of brain trauma, or … due to secondary complications or other complications

2. Vegetative State: Patients who remain unresponsive and speechless….

3. Severe Disability: The patient is conscious but needs the assistance of another person for some activities of daily living every day.…

4. Moderate Disability: Such a patient is able to look after himself at home, to get out and about to the shops and to travel by public transport. However, some previous activities, either at work or in social life, are now no longer possible by reason of either physical or mental deficit…

5. Good Recovery: This indicates the capacity to resume normal occupational and social activities, although there may be minor physical or mental deficits…social outcome should be included in the assessment here, such as leisure activities and family relationships

Wilson L, Boase K, Nelson LD, Temkin NR, Giacino JT, Markowitz AJ, Maas A, Menon DK, Teasdale G, Manley GT. A Manual for the Glasgow Outcome Scale-Extended Interview. J Neurotrauma. 2021 Sep 1;38(17):2435-2446. doi: 10.1089/neu.2020.7527. Epub 2021 Apr 6. PMID: 33740873; PMCID: PMC8390784

**Early Rehabilitation Barthel Index (ERBI) :**


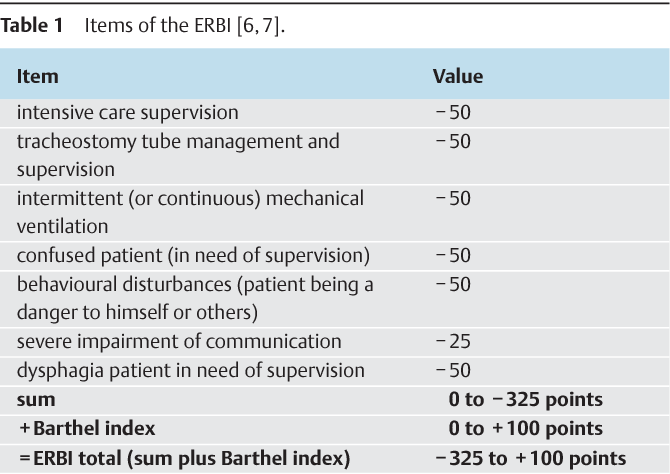


Rollnik JD. The Early Rehabilitation Barthel Index (ERBI). Rehabilitation 2011 ; 50 : 408-411

**Bogenhauser Dysphagia Score (BOD) :**

It is a German clinical swallowing examination.


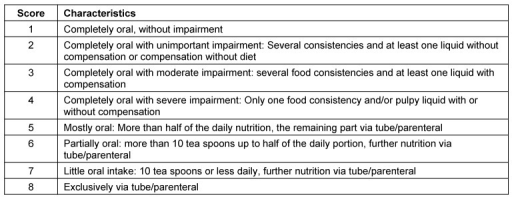


Arens C, Herrmann IF, Rohrbach S, Schwemmle C, Nawka T - GMS current topics in otorhinolaryngology, head and neck surgery (2015)

Dziewas R, Allescher HD, Aroyo I, Bartolome G, Beilenhoff U, Bohlender J, et al. Diagnosis and treatment of neurogenic dysphagia – S1 guideline of the German Society of Neurology. Neurol Res Pract. déc 2021;3(1):23.

**Functional Independance Measure (FIM) :**

**Motor Domain:**

**1. Self-care (6 items)**

– Eating
– Grooming
– Bathing
– Dressing – Upper body
– Dressing – Lower body
– Toileting

**2. Sphincter control (2 items)**

– Bladder management
– Bowel management

**3. Transfers (3 items)**

– Bed/Chair/Wheelchair
– Toilet
– Tub/Shower

**4. Locomotion (2 items)**

– Walk/Wheelchair
– Stairs

**Cognitive Domain:**

**5. Communication (2 items)**

– Comprehension
– Expression

**6. Social cognition (3 items)**

– Social interaction
– Problem solving
– Memory

Each item on the FIM is scored on a 7-point [Likert scale](https://strokengine.ca/en/glossary/likert-scale/), and the score indicates the amount of assistance required to perform each item (1 = total assistance in all areas, 7 = total independence in all areas)

[**https://strokengine.ca/en/assessments/functional-independence-measure-fim/**](https://strokengine.ca/en/assessments/functional-independence-measure-fim/)

**Level of cognitive functionning (LCF)**

It is the same scale than the RLAS (see above)

**Disability Rating Scale (DRS) :**

| **Category** | **Item** | **Instructions** | **Score** |
| --- | --- | --- | --- |
| **Arousability, Awareness and Responsivity** | Eye Opening | 0 = *spontaneous*  1 = *to speech*  2 = *to pain*  3 = *none* |  |
|  | Communication  Ability | 0 = *oriented*  1 = *confused*  2 = *inappropriate*  3 = *incomprehensible* 4 = *none* |  |
|  | Motor Response | 0 = *obeying*  1 = *localizing*  2 = *withdrawing*  3 = *flexing*  4 = *extending*  5 = *none* |  |
| **Cognitive Ability for Self Care Activities** | Feeding | 0 = *complete*  1 = *partial*  2 = *minimal*  3 = *none* |  |
|  | Toileting | 0 = *complete*  1 = *partial*  2 = *minimal*  3 = *none* |  |
|  | Grooming | 0 = *complete*  1 = *partial*  2 = *minimal*  3 = *none* |  |
| **Dependence on Others** | Level of Functioning | 0 = *completely independent*  1 = *independent in special environment*  2 = *mildly dependent*  3 = *moderately dependent*  4 = *markedly dependent*  5 = *totally dependent* |  |
| **Psychosocial  Adaptability** | Employability | 0 = *not restricted*  1 = *selected jobs*  2 = *sheltered workshop (non-competitive)*  3 = *not employable* |  |
| **Total DR Score** | | |  |

**Disability Categories**

| **Total DR Score** | **Level of Disability** |
| --- | --- |
| **0** | None |
| **1** | Mild |
| **2-3** | Partial |
| **4-6** | Moderate |
| **7-11** | Moderately Severe |
| **12-16** | Severe |
| **17-21** | Extremely Severe |
| **22-24** | Vegetative State |
| **25-29** | Extreme Vegetative State |

Rappaport et al., (1982). Disability rating scale for severe head trauma patients: coma to community. Archives of Physical Medicine and Rehabilitation, 63: 118-123

**Functionnal Dysphagia Scale (FDS) :**

| **Factor** | **Coded Value** | Empty Cell | **Score** |
| --- | --- | --- | --- |
| Lip closure | Intact | 0 | 10 |
|  | Inadequate | 5 |  |
|  | None | 10 |  |
| Bolus formation | Intact | 0 | 6 |
|  | Inadequate | 3 |  |
|  | None | 6 |  |
| Residue in oral cavity† | None | 0 | 6 |
|  | ≤10% | 2 |  |
|  | 10%-50% | 4 |  |
|  | ≥50% | 6 |  |
| Oral transit time* | ≤1.5s | 0 | 6 |
|  | >1.5s | 6 |  |
| Triggering of pharyngeal swallow | Normal | 0 | 10 |
|  | Delayed | 10 |  |
| Larynegal elevation and epiglottic closure | Normal | 0 | 12 |
|  | Reduced | 12 |  |
| Nasal penetration† | None | 0 | 12 |
|  | ≤10% | 4 |  |
|  | 10%-50% | 8 |  |
|  | ≥50% | 12 |  |
| Residue in valleculaeDagger; | None | 0 | 12 |
|  | ≤10% | 4 |  |
|  | 10%-50% | 8 |  |
|  | ≥50% | 12 |  |
| Residue in pyriform sinuses‡ | None | 0 | 12 |
|  | ≤10% | 4 |  |
|  | 10%-50% | 8 |  |
|  | >50% | 12 |  |
| Coating of pharyngeal wall after swallow | No | 0 | 10 |
|  | Yes | 10 |  |
| Pharyngeal transit time* | ≤1.0s | 0 | 4 |
|  | >1.0s | 4 |  |
| Total |  |  | 100 |

* Reference values from Logemann. † Relative percentage of the total bolus. ‡Relative percentage of the pertinent area on videofluoroscopic 2-dimensional view.

Han TR, Paik NJ, Park JW. Quantifying swallowing function after stroke: A functional dysphagia scale based on videofluoroscopic studies. Archives of Physical Medicine and Rehabilitation. mai 2001;82(5):677‑82.

**Penetration aspiration scale (PAS) :**

| **Score** | **Description of events** |
| --- | --- |
| 1 | Material does not enter airway |
| 2 | Material enters the airway, remains above the vocal folds, and is ejected from the airway |
| 3 | Material enters the airway, remains above the vocal folds, and is not ejected from the airway |
| 4 | Material enters the airway, contacts the vocal folds, and is ejected from the airway |
| 5 | Material enters the airway, contacts the vocal folds, and is not ejected from the airway |
| 6 | Material enters the airway, passes below the vocal folds, and is ejected into the larynx or out of the airway |
| 7 | Material enters the airway, passes below the vocal folds, and is not ejected from the trachea despite effort |
| 8 | Material enters the airway, passes below the vocal folds, and no effort is made to eject |

Alkhuwaiter M, Davidson K, Hopkins-Rossabi T, Martin-Harris B. Scoring the Penetration-Aspiration Scale (PAS) in Two Conditions: A Reliability Study. Dysphagia. 2022 Apr;37(2):407-416. doi: 10.1007/s00455-021-10292-6. Epub 2021 Apr 21. PMID: 33880656; PMCID: PMC8528890.

**Korean Mini Mental State Examination (K-MMSE) :**

It is the Korean translated version of the English MMSE


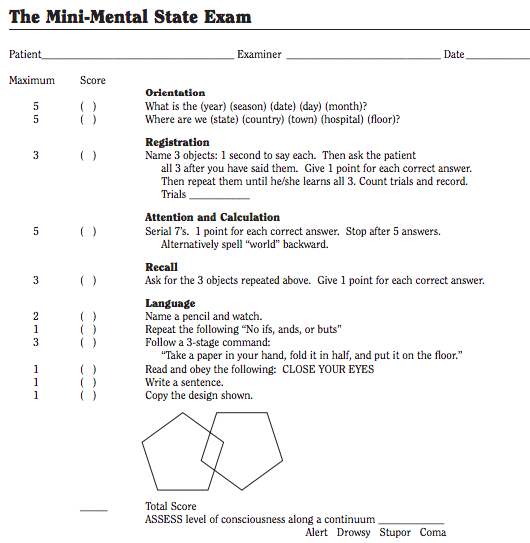


<https://cgatoolkit.ca/Uploads/ContentDocuments/MMSE.pdf>

**Korean Modified Barthel Index (K-MBI):**

This is a Korean translated version of the English MBI


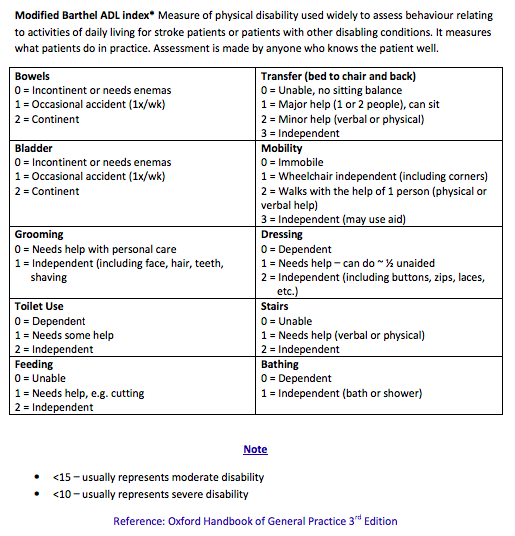


**National Institutes of Health Stroke Score (NIHSS):**

<https://www.ninds.nih.gov/health-information/public-education/know-stroke/health-professionals>

**Barthel Index:**

**
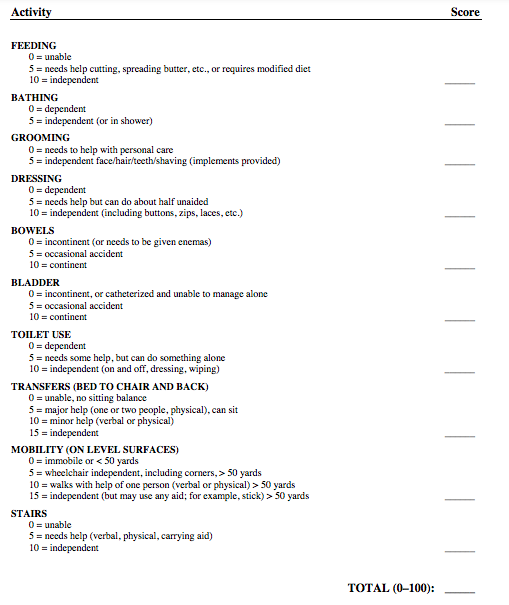
**

Mahoney FI, Barthel D. “Functional evaluation: the Barthel Index.” Maryland State Medical Journal 1965;14:56-61.

**Modified Rankin Scale (mRS):**

0 No symptoms at all

1 No significant disability despite symptoms; able to carry out all usual duties and

activities

2 Slight disability; unable to carry out all previous activities, but able to look after own

affairs without assistance

3 Moderate disability; requiring some help, but able to walk without assistance

4 Moderately severe disability; unable to walk without assistance and unable to attend to

own bodily needs without assistance

5 Severe disability; bedridden, incontinent and requiring constant nursing care and

attention

6 Dead

Rankin J. “Cerebral vascular accidents in patients over the age of 60.” Scott Med J 1957;2:200-15 Bonita R, Beaglehole R. “Modification of Rankin Scale: Recovery of motor function after stroke.” Stroke 1988 Dec;19(12):1497-1500

**Airway Care Score (ACS):**

Grading for the Airway Care Score

| **Grading** | **Cough to suction** | **Sputum quantity** | **Sputum character** | **Sputum viscosity** | **Suctioning frequency** |
| --- | --- | --- | --- | --- | --- |
| 0 | Vigorous | None | Clear | Watery | >3 h |
| 1 | Moderate | 1 pass | Tan | Frothy | Every 2-3 h |
| 2 | Weak | 2 passes | Yellow | Thick | Every 1-2 h |
| 3 | None | ≥3 passes | Green | Tenacious | <Every 1 h |

Tanwar G, Singh U, Kundra S, Chaudhary AK, Kaytal S, Grewal A. Evaluation of airway care score as a criterion for extubation in patients admitted in neurosurgery intensive care unit. J Anaesthesiol Clin Pharmacol. 2019 Jan-Mar;35(1):85-91. doi: 10.4103/joacp.JOACP_362_17. PMID: 31057247; PMCID: PMC6495608.

**Stroke-related Early Tracheostomy (SET) score:**


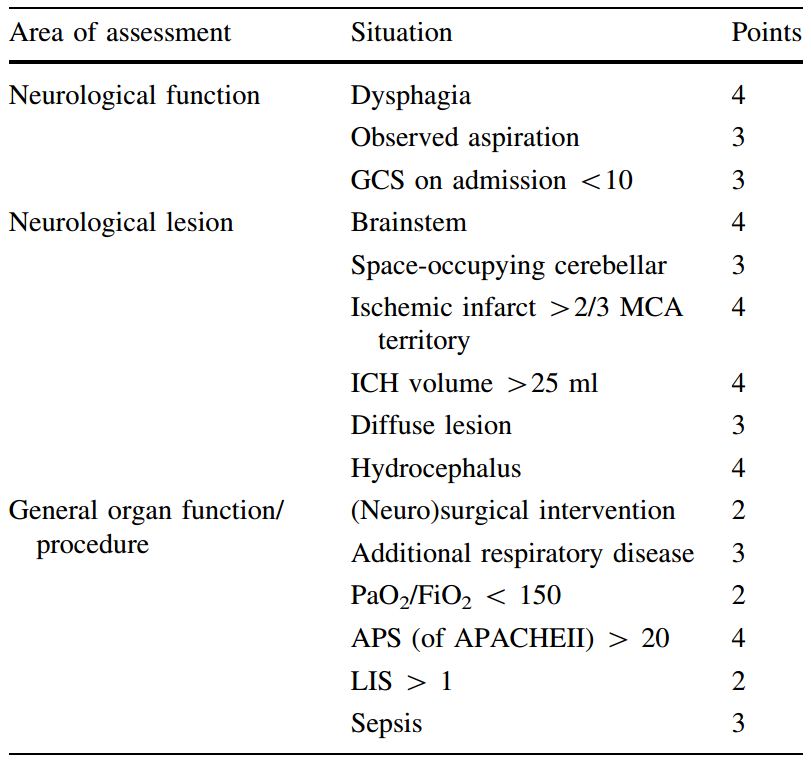


Schönenberger, Silvia et al. “The Setscore To Predict Tracheostomy Need In Cerebrovascular Neurocritical Care Patients”. *Neurocritical Care* 25.1 (2016): 94-104.

**World Federation of Neurosurgical Surgeons (WFNS):**


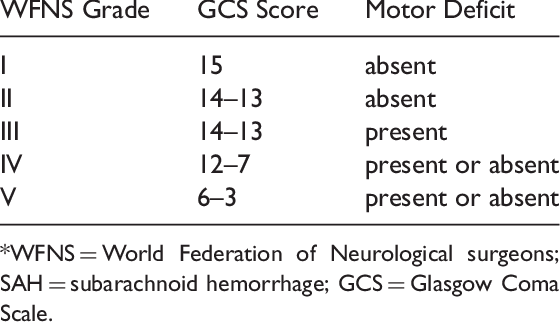


[Report of World Federation of Neurological Surgeons Committee on a Universal Subarachnoid Hemorrhage Grading Scale. J Neurosurg. 1988 Jun;68(6):985-6.](https://doi.org/10.3171/jns.1988.68.6.0985)

Fisher Score:


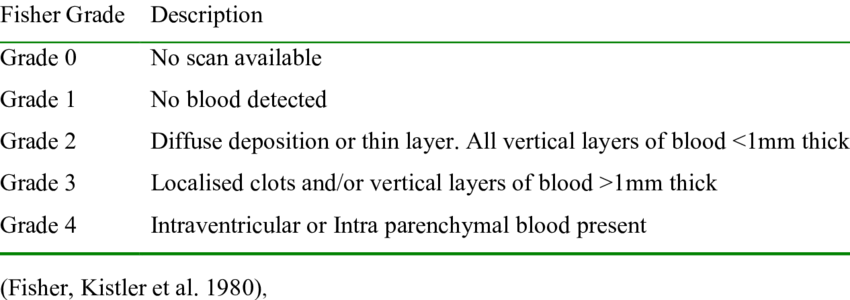


Fisher, C. M. M.D.; Kistler, J. P. M.D.; Davis, J. M. M.D.. Relation of Cerebral Vasospasm to Subarachnoid Hemorrhage Visualized by Computerized Tomographic Scanning. Neurosurgery 6(1):p 1-9, January 1980.
